# The Influence of Single Whole-Body Cryostimulation on Cytokine Status and Oxidative Stress Biomarkers during Exhaustive Physical Effort: A Crossover Study

**DOI:** 10.3390/ijms24065559

**Published:** 2023-03-14

**Authors:** Alicja Jurecka, Alina Woźniak, Celestyna Mila-Kierzenkowska, Beata Augustyńska, Łukasz Oleksy, Artur Stolarczyk, Artur Gądek

**Affiliations:** 1 Department of Orthopedics and Physiotherapy, Faculty of Health Sciences, Jagiellonian University Medical College, 30-688 Krakow, Poland; 2 Department of Medical Biology and Biochemistry, Ludwik Rydygier Collegium Medicum in Bydgoszcz, Nicolaus Copernicus University in Torun, 85-092 Bydgoszcz, Poland; 3 Institute of Physical Education, Kazimierz Wielki University, 85-091 Bydgoszcz, Poland; 4 Department of Physiotherapy, Faculty of Health Sciences, Jagiellonian University Medical College, 30-688 Krakow, Poland; 5 Department of Orthopedics and Rehabilitation, Medical University of Warsaw, 02-203 Warsaw, Poland

**Keywords:** skeletal muscles, exercise, cryostimulation, cytokine, myokines, reactive oxygen species, antioxidants

## Abstract

The purpose of the study was to assess the impact of single whole-body cryostimulation (WBC) preceding submaximal exercise on oxidative stress and inflammatory biomarkers in professional, male athletes. The subjects (*n* = 32, age 25.2 ± 37) were exposed to low temperatures (−130 °C) in a cryochamber and then participated in 40 min of exercise (85% HRmax). Two weeks afterwards, the control exercise (without WBC) was performed. Blood samples were taken before the start of the study, immediately after the WBC procedure, after exercise preceded by WBC (WBC exercise) and after exercise without WBC. It has been shown that catalase activity after WBC exercise is lower in comparison with activity after control exercise. The interleukin 1β (IL-1-1β) level was higher after control exercise than after WBC exercise, after the WBC procedure and before the start of the study (*p* < 0.01). The WBC procedure interleukin 6 (IL-6) level was compared with the baseline level (*p* < 0.01). The level of Il-6 was higher both after WBC exercise and after control exercise compared with the level recorded after the WBC procedure (*p* < 0.05). Several significant correlations between the studied parameters were shown. In conclusion, the changes in the cytokine concentration in the athletes’ blood confirm that body exposition to extremely low temperatures before exercise could regulate the inflammatory reaction course and secretion of cytokines during exercise. A single session of WBC in the case of well-trained, male athletes does not significantly affect the level of oxidative stress indicators.

## 1. Introduction

Over the last few years, we have deepened our knowledge regarding the biochemical consequences of exercise-induced oxidative stress. Although releasing high levels of reactive oxygen species (ROS) during exercise can harm cellular components, low levels of ROS play multiple regulatory roles in cellular processing, such as controlling gene expression, regulating signaling pathways, and modulating the production of the skeletal muscle force [1,2]. It has also been shown that the inhibition of ROS production blocks the effects of cytokine activity, including the expression of adhesive proteins and decrease in the permeability of the walls of capillaries [3]. On the other hand, a significant increase in ROS generation blocks the proliferation and differentiation of satellite cells in the location of the micro-injury [4].

It is well known that the immune response to the physical effort manifests itself in the concentration change in pro- and consequently anti-inflammatory cytokines, as well as in growth factors in the blood. The level of cytokine release resulting from a single instance of exercise depends on its intensity and duration, the area of active muscle groups and, last but not least, the nature of muscle work [5,6]. The acute inflammatory response of the organism to physical exercise is initiated by an increase in the number of migrating neutrophils present in the damaged muscle fibers up to 24 h after exercise. The function of the activators of neutrophils is performed, i.e., by proinflammatory cytokines (myokines), which include interleukin beta1 (IL-1β) and tumor necrosis factor alpha TNF-α. It has been indicated that these myokines also stimulate nicotinamide adenine dinucleotide phosphate (NADPH) oxidase, which catalyzes the generation of ROS and induces changes in the expression of the genes responsible for the synthesis of acute-phase proteins [7,8,9]. Macrophages also penetrate the site of inflammation, but the period of their activity is delayed from the time of injury [8,9]. While migrating to damaged skeletal muscle fibers, phagocytic cells not only remove the pieces of muscle tissue, but they also serve as a source of growth factors, e.g., transforming growth factor beta1 TGF-β, taking part in the regeneration of the damaged tissue. The platelet-derived growth factor (PDGF), in turn, as well as IL-6, is released by progenitor, mononuclear satellite (stem) cells of skeletal muscles, stimulating their proliferation and differentiation into multinucleated myocytes, which leads to the reconstruction of damaged muscle fibers [9].

Research conducted in recent years indicates that it is essential to maintain a physiologically low level of ROS for the proper course of several biological processes, such as the cell cycle, the immune response and the expression of many genes, including genes encoding cytokines [10]. The expression of cytokine genes is initiated by nuclear factor-κB (NF-κB) and activator protein 1 (AP-1) transcription factors, which are controlled by ROS [5,10]. It is believed that the activation of NF-kB during skeletal muscles work underlies the body’s adaptation to physical effort [7]. This adaptation to physical exercise may also be modified by mitochondrial activity, due to its role in bioenergetics processes, thermogenesis, and oxidative stress regulation. In their systematic review, Lim et al. [11] concluded that exercise training improves the mitochondrial oxidative capacity of both skeletal muscles and platelets in patients with cardiovascular diseases. In studies by Lippi et al. [12] on the effect of physical exercise on muscle mitochondria modifications in older adults, the improvement of mitochondrial density and dynamics by resistance training and the improvement of mitochondrial antioxidant capacity by endurance training were reported based on five databases (PubMed, Scopus, Web of Science, Cochrane, and PEDro). On the other hand, regular exercise training seemed not to represent a major stimulus of uncoupling protein 1 expression in the mitochondria of classical brown adipose tissue (CBAT) in animals [13]. However, regular exercise training was proven to induce adaptive responses to thermogenic activity of CBAT in animals, especially if the exercise was combined with cold exposure [13]. Exercise-induced effects on the mitochondrial life cycle may be related to an increase in the activity of peroxisome proliferator-activated receptor-γ coactivator-1α (PGC-1α), which is a potent mitochondrial respiration and biogenesis stimulator [14]. PGC-1α is also crucial in linking stimuli such as cold to an internal metabolic response such as mitochondrial biogenesis [15]. In addition, PGC-1α activity can be induced by ROS and plays a crucial role in the ROS homeostatic cycle [14].

The presence of ROS at the site of myocyte damage indirectly affects the reconstruction of the muscle fibers by inducing the proteasomes responsible for the degradation of the protein molecules (especially actin) [16,17]. Thus, the possibility of activating the mechanisms that regulate cytokine expression, which form part of the correct inflammatory process and maintain the physiological concentration of ROS during intensive exercise, when the oxidant–antioxidant homeostasis may be disturbed, seems to be of great importance. It is for this reason that it is justified to attempt to find the stimuli that modulate the inflammatory reaction through the activation of the antioxidative mechanisms of the organism. Cryogenic stimulation (cryostimulation) seems to be one of the methods of stimulating athletes’ organism to cope with larger training loads, as well as to prevent traumas and physical stress symptoms [17,18,19,20].

Cryostimulation is a method of physiotherapy (in the field of physical therapy methods) based on the organism’s reaction to stimulation by low temperatures. The basic idea of cryostimulation is to cool the body tissues in the fastest way possible to provoke active hyperemia [21]. From the therapeutic point of view, these reactions are beneficial and effective in restoring the natural balance of the human body. Until recently, cryostimulation was predominantly used in treating several diseases, e.g., inflammatory diseases of locomotor systems, degenerative diseases, rheumatic diseases, autoimmunological diseases, sclerosis multiplex, osteoporosis, fibromyalgia and many more [22]. However, the beneficial effects of cryostimulation, including better biological regeneration, muscle recovery from physical workout, as well as prevention from overload of the locomotion system, make this method increasingly useful in sports medicine [23,24]. Recently, whole-body cryostimulation (WBC) has become a popular mode of cryostimulation. Very few studies indicate the possibility of using cryostimulation as an effective method of prevention in exercise-induced skeletal muscle injury, by the regulation of oxidative and inflammatory processes [17,18,25].

Whole-body cryotherapy (cryostimulation) means that the whole body surface is stimulated by extremely low temperatures (below −100 °C) in a cryochamber. The entire process lasts less than 2–3 min [21]. The positive effects of WBC for sports medicine treatments have been indicated. This type of cryostimulation causes active peripheral hyperemia, which results in improved metabolism and fast elimination of harmful metabolism products (especially lactate and histamine). Cryostimulation reduces algesthesis and a decrease in skeletal myotonus leads to increased muscle strength. The WBC boosts the hypothalamus–hypophysis hormone axis and, as a result, increases the plasmatic concentration of glucocorticosteroids, adrenaline, testosterone and gonadotrophins [26]. Some authors emphasize the beneficial effect of cryostimulation on the antioxidant system [23,24,27,28,29,30]. However, data found in the literature mainly concern prolonged WBC treatment with different numbers of sessions during a vigorous training cycle [18,26,27,30].

Because cytokines regulate various processes, i.e., the immune response to exercise, cell proliferation and differentiation, several studies published in recent years indicate the effect of exercise and WBC on the levels of these molecules. The most commonly determined cytokines in this research were as follows: Il-1, interleukin 6 (Il-6), interleukin 10 (Il-10), and TNF-α [19,31]. Moreover, our own preliminary studies conducted among a small group of athletes indicate the participation of ROS in the release of cytokines into the bloodstream in response to exercise and cryostimulation [24]. This became the basis for the continuation of the research in this area to enable a clear interpretation of the ways in which extremely low temperatures affect free radical processes and simultaneously affect the regulation of the inflammation that occurs during a single physical event.

The scientific literature lacks high-quality methodological articles on the impact of pre-exercise WBC as a method that could potentially modulate immunological and oxidative reactions during exhaustive physical effort. This experiment aimed to examine whether the application of this particular WBC procedure before exhaustive exercise may cause changes in the concentration of selected cytokines (IL-1β, IL-6, TNF-α and TGF-β1), as well as superoxide dismutase (SOD), catalase (CAT), glutathione peroxidase (GPx) and creatine kinase (CK) activity, the level of antioxidant vitamins (A and E vitamins) and lipid peroxidation products (conjugated dienes (CD), thiobarbituric acid reactive substances (TBARS)) and total lipid peroxides (LOOH) concentration in the blood of well-trained male athletes. These parameters are reliable indicators of exercise-induced damage to myocytes and play a significant role in the organism’s reaction to extremely low temperatures.

## 2. Results

### 2.1. Basic Characteristics of the Study Group

The chosen anthropometric characteristics and physiological parameters of the studied group are presented in Table 1. The intensity of workouts during the last 7 days before the study was moderate (4 METs), with a mean of 3733 METs per week, while the degree of physical activity in the previous week of the competitive season was defined as severe (8 METs), with a mean of 9636 METs per week.

The sportsmen evaluated their exercise during the experiment (both after the first and the second session) as “severe” according to the Borg CR10 scale. The difference in Borg CR10 scores between session 1 and session 2 was statistically insignificant (Table 1).

No statistically significant differences were observed between the physical working capacity 170 (PWC170) test (*p* > 0.05) and the maximum rate of oxygen consumption (VO_2_ max, *p* > 0.05) values obtained before session 1 and before session 2 (Table 1).

### 2.2. The Level of Oxidative Stress Biomarkers

The largest effect size in the sample was observed for CAT (η^2^ = 0.124). A single cryostimulation procedure did not per se significantly influence CAT activity in the subjects’ erythrocytes (Table 2). However, CAT activity measured after the exercise preceded by an exposition of cryogenic temperatures was 24% lower (*p* < 0.001) compared to the control exercise (Table 2). It is also significant that the CAT activity measured after the exercise preceded by WBC did not change in comparison with the activity measured during the rest period (before the start of the study). On the other hand, the activity of the enzyme measured after exercise without cryostimulation was 29% higher than the CAT activity measured before the start of the experiment (*p* < 0.01). No statistically significant CAT activity was observed in the sportsmen’s erythrocytes after the exercise connected with WBC concerning the CAT activity measured directly after the procedure. However, after the exercise without WBC, the activity of these enzymes was 29% higher (*p* < 0.01) when compared with the CAT activity measured directly after cryostimulation (Table 2). No statistically significant changes in SOD activity were observed in the blood of the subjects after a single session of WBC and/or exercise. When comparing the activity preceded by cryostimulation and without it, it was observed that the SOD activity in erythrocytes was insignificantly lower when the exercise was linked with WBC (Table 2). No statistically significant differences were observed when comparing GPx activity in the athletes’ blood before entering and after leaving the cryo-chamber. In addition, no differences were noticed when comparing the activity of this enzyme after exercise preceded and not preceded by cryochamber stimulation. However, GPx activity after the exercise not connected with WBC was 26% higher than its activity preceded by WBC (*p* > 0.05) (Table 2). In the blood plasma of the sportsmen, no statistically significant changes in the concentration of A and E vitamins were found after cryotherapy or after exercise in either condition (Table 2).

From a statistical point of view, no important changes in the level of CD, LOOH or TBARS occurred in the blood of the sportsmen who underwent WBC and/or intensive exercise. However, the CD level in the serum tested after a single session of WBC showed a tendency to drop when compared to the level tested before the therapy (Table 3). The concentration of TBARS and LOOH in the serum was lower after the WBC exercise than after the control exercise (*p* > 0.05).

### 2.3. The Cytokine Concentration and CK Activity

CK activity in the subjects’ serum did not undergo statistically significant changes either directly after a single session of WBC nor after the exercise preceded by WBC or control exercise (Table 3). However, there was a tendency for CK activity to lower after WBC. When comparing CK activity after the exercise linked with cryostimulation and after the control effort, slightly lower activity was reported if the activity was preceded by WBC (*p* > 0.05).

The most significant effect size in the sample was recorded for IL-1β (η^2^ = 0.277) and IL-6 (η^2^ = 0.333). The statistical analysis showed a higher concentration of IL-1β in serum after the exercise without WBC compared with the concentration measured before a single session of WBC (*p* < 0.01). The concentration of IL-1β was 48% higher compared to the baseline concentration (*p* < 0.05, Table 3). The concentration of IL-1β after control exercise was 45% higher than the concentration measured directly after WBC (*p* < 0.01, Table 3). Comparing both exercise conditions, the level of IL-1β was more than two times lower when the exercise was preceded by WBC. Single whole-body cryostimulation and/or exercise did not result in statistically significant changes in TNF-α concentration in the athletes’ blood. However, a tendency for the TNF-α concentration to drop after exercise with cryostimulation was observed in comparison with the concentration of this cytokine measured before the start of the study (Table 3). A single WBC procedure caused a 52% drop in the Il-6 concentration in serum (*p* < 0.01, Table 3). After exercise preceded by WBC, the concentration of Il-6 in the blood rose by 95% compared to the concentration of this interleukin when measured immediately after WBC (*p* < 0.05, Table 3). The level of Il-6 was 162% higher after control exercise in comparison with the concentration measured after the WBC procedure (*p* < 0.001, Table 3). However, when comparing WBC exercise and control exercise, no statistically significant differences were noted regarding the Il-6 concentration in the serum of the sportsmen. No statistically significant differences were noted for the TGF-β1 concentration comparing all study conditions (Table 3).

### 2.4. Correlations between the Studied Parameters

Several significant correlations between the studied parameters were shown. Negative correlations were observed between the concentration of TNF-α in serum and TBARS in plasma (r = −0.518; *p* < 0.05) and erythrocytes (r = −0.799; *p* < 0.001) before a single session of WBC. At this point of the test, a negative correlation was also observed between the concentration of TGF-β in serum and TBARS in plasma (r = −0.617; *p* < 0.05). Statistically significant correlations were observed after WBC between the level of TNF-α and LOOH (r = 0.717; *p* < 0.01) and between the levels of TGF-β1 and LOOH (r = −0.544; *p* < 0.05) in serum. 

**Figure 1:**
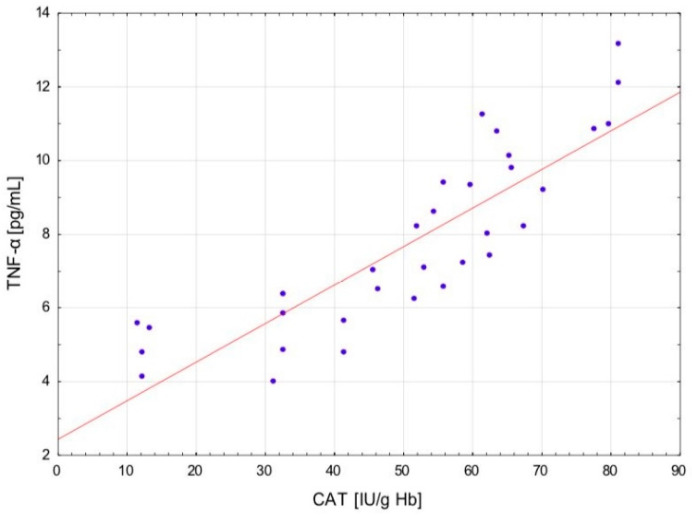
Linear regression (r = 0.86; *p* < 0.001) of catalase (CAT) activity versus tumor neurosis factor alpha (TNF-α) concentration in the blood of sportsmen after exercise preceded by single whole-body cryostimulation (WBC).

**Figure 2:**
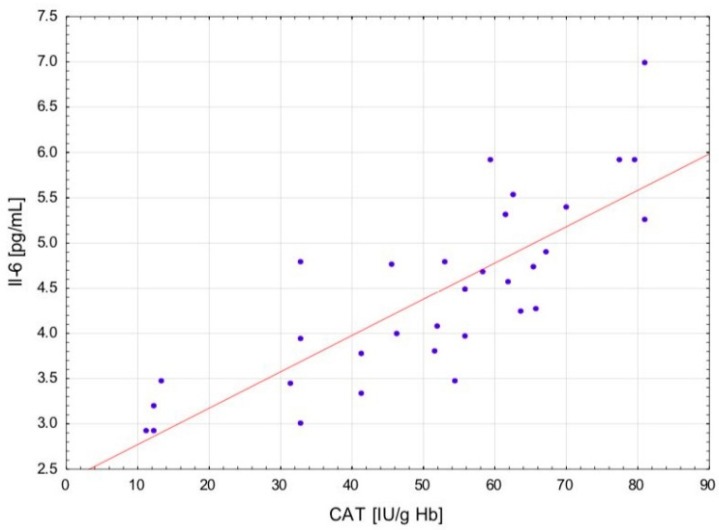
Linear regression (r = 0.81; *p* < 0.001) of catalase (CAT) activity versus interleukin- 6 (IL-6) concentration in the blood of sportsmen after exercise preceded by single whole-body cryostimulation (WBC).

**Figure 3:**
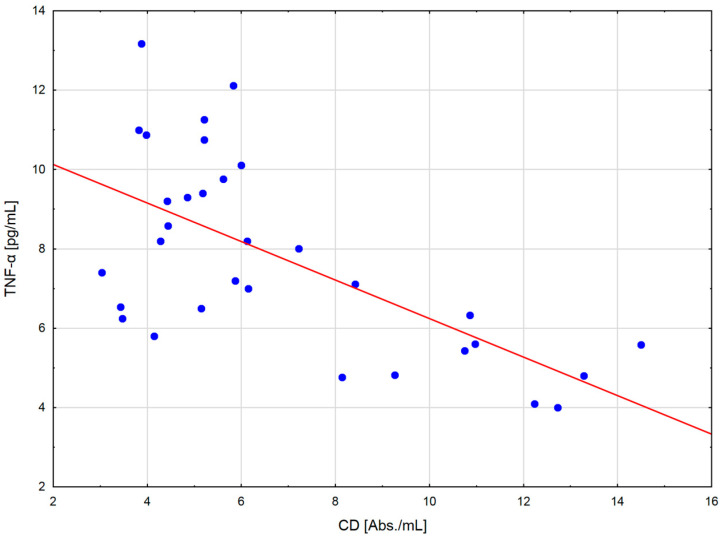
Linear regression (r = −0.64; *p* < 0.001) of conjugated dienes (CD) level versus tumor neurosis factor alpha (TNF-α) concentration in the blood of sportsmen after exercise preceded by single whole-body cryostimulation (WBC).

**Figure 4:**
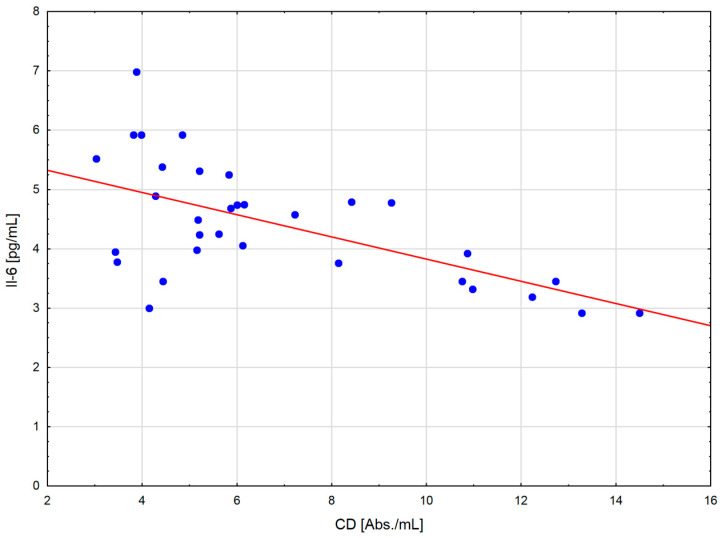
Linear regression (r = −0.538; *p* < 0.05) of conjugated dienes (CD) level versus interleukin-6 (IL-6) concentration in the blood of sportsmen after exercise preceded by single whole-body cryostimulation (WBC).

After the exercise preceded by a single session of WBC, a positive correlation occurred between the levels of LOOH and TNF-α (r = 0.721; *p* < 0.01) and Il-6 (r = 0.658; *p* < 0.05) in serum, as well as between the CAT activity in erythrocytes and the concentration of TNF-α (r = 0.86; *p* < 0.001) (Figure 1) and IL-6 (r = 0.81; *p* < 0.001) in serum (Figure 2). In addition, at the same time, negative correlations were observed between the CD levels in plasma and TNF-α (r = −0.64; *p* < 0.001) (Figure 3), Il-1β (r = −0.538; *p* < 0.05) and Il-6 (r = −0.61; *p* < 0.001) (Figure 4) in serum, as well as between the level of vitamin A in plasma and the concentration of TNF-α (r = −0.583; *p* < 0.05) and Il-6 (r = −0.606; *p* < 0.05) in serum. After the physical exertion preceded by WBC, a negative correlation manifested between the vitamin A levels in plasma and LOOH levels in serum (r = −0.716; *p* < 0.01). After the control exercise, a positive correlation was observed between vitamin A and CD in plasma (r = 0.648; *p* < 0.05).

## 3. Discussion

The findings confirm that WBC may regulate the oxidant–antioxidant balance (OAB) during exercise/sports training. Lubkowska et al. [32] observed a total oxidative status decline, demonstrating statistical importance, in the 30 min following the exit from the cryochamber, as well as a drop in the total antioxidative status of the blood serum of young men who did not undergo any form of cryotherapy/cryostimulation prior to this. The authors assume that these results point to the decreased ROS generation as an effect of body exposure to cryogenic temperatures or to a significant role of the non-enzymatic system in removing the oxygen free radicals (FOR) generated during WBC. Earlier studies on weight-lifters and amateurs who used a protocol similar to this study also showed a lowering of the TBARS concentration in erythrocytes of weight-lifters, as well as a tendency for the CD and TBARS levels to drop in the plasma of weight-lifters and amateurs directly after a single session of WBC [17].

Our own studies show that a single WBC session in the case of the professional, male athletes is not a source of oxidative stress. In contrast, after the procedure, no statistically significant changes in the oxidative indicators of lipid damage levels were observed neither in erythrocytes nor in the plasma of the subjects. However, some tendencies for a decrease in the start and end products of peroxidation in the blood immediately after leaving the cryochamber have been reported, so this may suggest the beneficial influence of even a single session of WBC on the OAB of the organism. A similar comparison of the exercise preceded by cryotherapy and without it showed that despite an additional potential source of ROS generation in the form of cryogenic temperatures, the oxidative damages to myocytes after the exercise preceded by WBC were slightly lower than without the cryostimulation.

An alteration in antioxidant enzyme action and the accumulation of low molecular non-enzymatic antioxidants result from the increased exposition of cells to the reactive forms of oxygen and nitrogen during active skeletal muscle work. FOR are generated during exercise as regulators of the expression of genes that code the proteins responsible for the antioxidative defense of the organism. It has also been proven that ROS may directly or indirectly influence the activity of some transcription factors, and thus induce the biosynthesis of some of the proteins in the body [33].

Although ROS are commonly viewed as a harmful side-product of oxygen metabolism, increased ROS levels during exercise can also contribute to some post-exercise adaptations [30,34], which seems highly likely in the studied group of trained players of sports clubs (volleyball, basketball and football). Moderate exposure to ROS is necessary to trigger the organism’s adaptive responses, such as the activation of the antioxidant defense mechanism [35,36].

Nowadays, it is known that their generation is also controlled by using their signaling and regulatory functions. ROS comprise a stimulus for cells to transform as a response to physiological stress [33]. During exercise, when muscle fiber is damaged, ROS initiate the process of the regeneration of the damaged myocytes. Although they are per se responsible for damaging the cells, their generation may be a secondary phenomenon, not linked with the initial mechanism of the damage, which occurs during the inflammatory reaction. In this case, ROS act as a mediator for the repair processes [37,38]. This is why the activation of the antioxidative mechanism that maintains the physiological concentration of ROS during increased physical activity, when the oxidative–antioxidative homeostasis is disrupted, seems particularly significant. 

Our own studies conclude that a single session of WBC did not per se change the antioxidant enzymes’ activity in the erythrocytes of well-trained athletes. CAT activity after the exercise linked with WBC did not change compared to its activity during rest, but it statistically significantly rose after exercise with WBC in comparison to the enzyme activity before the experiment onset. Furthermore, it was confirmed that the enzymatic activity in erythrocytes when measured after the exercise preceded by an exposition to cryogenic temperatures was statistically significantly lower than that observed after the control exercise. The activity of SOD and GPx was higher in the erythrocytes when the exercise was not preceded by cryostimulation. However, this difference was not statistically significant.

Erythrocytes are prone to damage caused not only by the activity of ROS in the cell’s surroundings, but also as a result of ROS generation inside the cell due to the intensification of oxyhemoglobin in the methemoglobin autoxidation process. The anion-radical superoxide is dismutated to hydrogen peroxide. By reacting with transition metals, H_2_O_2_ participates in the generation of the hydroxyl radical, which is responsible for initiating the lipid oxidation process [39]. Because of the intensified peroxidation of membrane lipids, the hemolysis of red cells increases and the hem and iron released due to the denaturation of hemoglobin become the following factors in the generation of FOR [40]. The lack of change in CAT activity in well-trained athletes after the exercise connected with WBC and the concurrent increase in the activity of this enzyme after the exercise without stimulation by extremely low temperatures, compared to its activity measured before the start of the study, possibly shows the limited extent of the oxidation damage to the erythrocytes as an effect of exercise. The lack of an increase in antioxidative enzyme activity after the exercise with WBC may confirm that fewer ROS are generated.

WBC activates the hypothalamus–pituitary hormonal axis, which increases the plasma hormones in the blood. Single cryochamber stimulation causes a significant increase in the concentration of catecholamines in the blood [41]. It is believed that adrenalin may mediate ROS generation, as well as limit their generation by neutrophils [42]. The fact that adrenaline limits ROS generation may explain the lower CAT activity in the blood of well-trained, male athletes after the exercise preceded by single systemic cryotherapy, when compared to its activity after the control exercise. The regeneration process of the myocytes damaged during exercise stimulates the phagocytic cells, which gather at the damage site. Moreover, they are another source of ROS [6,37,43,44]. 

A single session of WBC performed on untrained men caused not only an increase in SOD, but also increased the activity of GPx located in erythrocytes. A concurrent increase in CD concentration occurred in plasma as well as in erythrocytes [27]. A single exposure to cryogenic temperatures caused a rise in the activity of glutathione reductase and peroxidase with a simultaneous decrease in CAT activity and glutathione transferase in the erythrocytes of young, healthy men 30 min after the therapy [23]. The quoted findings [23] may indicate a specific limitation of our own research related to the timing of sample collection. During our own study, blood samples were collected only immediately after the end of the WBC procedure, as well as immediately after the end of the exercises (both in the case of session 1 and session 2).

The specific period of the experiment should also be noted, which may be a limitation of our own research. All procedures related to the experiment were carried out on professional athletes during the recovery period after the end of sports competitions. At the same time, however, a potential source of oxidative stress in the form of daily sports training was excluded. This could have been significant in relation to the results observed in our own research, mostly the statistically insignificant changes in the level of lipid peroxidation products, or the activity of antioxidant enzymes.

Some non-enzymatic free radical scavengers also participate in the organism’s defense mechanism from oxidative damage. In our own study, we noticed no statistically significant changes in the vitamin A and E concentration in the blood of well-trained athletes and these results generally confirm the research by other authors [24,30]. Currently, little is known about the influence of WBC on the concentration of anti-oxidative vitamins in the blood. A slightly higher concentration of the tested vitamins in the blood of the professional, male athletes after the exercise preceded by an exposition to extremely low temperatures observed in this study, compared to control exercise, with slightly lower activity of antioxidative enzymes and lower concentration of lipid peroxidation products, suggests that even a single session of WBC may limit the oxidative damages to lipids. A confirmation of the role of the tested vitamins in protecting the phospholipids of the cell membranes of myocytes, as well as protecting erythrocyte membranes from ROS attack, can also be found in the statistically significant negative correlations between the levels of the vitamins and the lipid peroxidation products in the blood of well-trained sportsmen after the exercise preceded by WBC.

It seems that oxidative stress is necessary to improve physical performance as a result of sports training [34,45]. Intensive exercise can, on the one hand, cause DNA damage and the oxidation of lipids and proteins [46], and on the other hand, activate the endogenous antioxidant defense mechanisms of the system [47,48]. The phenomenon of the beneficial effect of small doses of an agent that is harmful in larger doses is called hormesis (gr. to set in motion). Thus, it seems that the increased ROS generation induced by both exercise [49,50,51,52] and WBC [23,27,28,29,30] is part of the hormesis phenomenon. It has been shown that repeated exposure to a factor that causes mild oxidative stress stimulates proteasomes, which contributes to the accelerated removal of oxidatively damaged proteins, reduces the sensitivity of isolated cells to the destructive effects of ethanol and hydrogen peroxide, as well as activates enzymatic antioxidant mechanisms, resulting in a delay in the aging process [53]. It is possible that the mostly statistically insignificant changes in the examined oxidative stress indicators after WBC and/or submaximal physical effort observed in our own research are associated with the constant presence of elevated FOR levels in professional athletes during training. The results of our own study are in accordance with other investigations, in which after exercise, regardless of its nature (aerobic or anaerobic), no significant increase in oxidative stress biomarkers was observed in trained people [34,54,55]. It is also worth noting that most of the studies indicated the lack of post-exercise changes, mostly in the level of by-products of aerobic metabolism [56,57,58]. The cited results of our own and other authors’ research [34,54,55] are probably related to the reduction in ROS generation as a form of adaptation to regular exercise and/or a protective effect of the antioxidant system in response to a stress factor [34]. These findings may be related to the increased activity of specific repair enzymes [34,59], the activity of which was not determined in the presented study.

The lack of changes in the concentration of lipid peroxidation products observed in our own research may also result from the increased rate of their removal in well-trained athletes (footballers, volleyball players and basketball players) who have adapted to long-term physical endurance activities [18]. The level of TBARS, CD and LOOH in the blood is the resultant of their production and removal processes from damaged tissues [18,60]. This fact is consistent with the demonstrated increase in CAT activity and the tendency to increase SOD and GPx activity in the tested athletes after exercise. Antioxidant enzymes are the first line of defense against ROS [61,62,63], so it is possible that they may have sufficiently limited the formation and/or impact of reactive radical species on tissues in the well-trained athletes. Antioxidant vitamins are another line of defense, leading to the interruption of free radical oxidation reactions [63,64]. Presumably, the lack of changes in the level of vitamins A and E after exercise, observed in our own research, is related to the efficiently functioning enzymatic antioxidant defense mechanism in this group of athletes, which is the first to react in the event of oxidative–antioxidant imbalance. However, it also seems highly probable that the demonstrated lack of changes in the antioxidant vitamins level may result from blood condensation after exercise associated with water loss [65]. In the experiment, blood samples were collected once, immediately after exercise (both during session 1 and session 2).

A specific period of the study, attributable to the recovery time after the end of the training–competition cycle, could have affected the results obtained. During the experiment, the subjects were, therefore, deprived of additional sources of ROS that may appear in the course of preparation for the competition, i.e., stress related with the upcoming start or intensive training. 

There is a strong evidence that both training as well as a single session of physical activity may cause changes in the immunological system that are linked, i.e., with the number of leucocytes, the properties of granulocytes, the concentration of immunoglobulin A and C-reactive protein (CRP), as well as many pro- and anti-inflammatory cytokines [6,16,44,66,67].

An acute inflammatory reaction is initiated by the increase in the number of migrating neutrophils present in the damaged muscles for up to 24 h after exercise. Pro-inflammatory cytokines (myokines, for example IL-1β and TNF–α) play the role of neutrophil activators. It has been shown that these myokines also activate NADPH oxidase, which catalyzes the generation of ROS, and induces changes in the expression of the genes responsible for the synthesis of the acute-phase proteins [8,9]. Moreover, macrophages permeate into the location of the inflammation, but the period of their activity is delayed in relation to the time when the damage has occurred [9]. Phagocyte cells that migrate to the damaged skeletal muscle fibers remove fragments of the muscle tissue and are also a source of growth factors, for example TGF-β, which participates in the regenerative processes of the cell. The PDGF and Il-6 generated by the progenitor one-nucleus satellite (stem) cells of the skeletal muscles stimulate their proliferation and differentiation into multi-nucleus myocytes, which as a result leads to the regeneration of the damaged muscle fibers [9,37].

Thus, the response of the immunological system is visible in the change in the concentration of the pro- and anti-inflammatory cytokines, as well as growth factors, in the blood. It is commonly known that the level of the cytokines generated after exercise depends on its intensity and duration. The localization of the area of the active muscles is also important [68]. Prolonged endurance training causes the most severe damages in the area of the skeletal muscle fibers and the tissue surrounding them. Taking part in a marathon causes the levels of TNF-α and IL-1β to multiply by 2–3 times and the concentration of IL-6 in blood plasma even by 100 [6]. Moreover, a high level of IL-6 in the blood may be viewed as an early, sensitive marker of inflammatory processes.

Thomas et al. [69] exposed runners and weightlifters to specialized training and they showed that both types of training induced a slight rise in the level of Il-6 in the sportsmen’s blood. However, it did not induce an acute inflammatory reaction, as there were no changes in CRP directly after the exercise. There are also some suggestions that the rise in Il-6 that happens after exercise is not directly linked with the myocyte damage caused by the exercise [70]. This increase in pro-inflammatory cytokines in blood plasma has been observed by Nemet et al. [67] in the case of weightlifters 14 days after training.

It is thought that the profile of cytokines after exercise/training is significantly influenced by the ratio of the aerobic component to the anaerobic metabolism, the area of the muscle tissue that is suffering from increased blood redistribution, as well as the type of active muscles fibers. It has been shown that the expression of the gene TNF-α, interleukin-15 and interleukin-18 is elevated in type II muscle fibers (quickly contracting). The same can be said about type I (slowly contracting) muscle tissue [71].

Our own studies have shown a significant increase in IL-1β in professional athletes’ serum following control exercise compared to the concentration measured during the rest period. However, the level of Il-6 in the case of well-trained sports club players after exercise on a cycloergometer showed a slight tendency to rise when compared to the concentration of this interleukin measured before the start of the study. From a statistical point of view, the exercise did not induce any major changes in the concentration of TNF–α in the subjects’ blood.

The increase in the level of IL-1β in the blood that is statistically important and was shown in this study after a 40 min session of submaximal exercise is probably linked with the chemotactic properties of this interleukin concerning neutrophils and monocytes. By activating phagocytic cells as well as proteolytic enzymes, IL-1β participates in the regeneration of the skeletal muscle fibers that have been damaged during exercise. A heightened level of IL-1β in the blood also increases the permeability of the mesothelium and stimulates ROS generation [72].

The studies conducted in recent years have shown that maintaining a physiological, low level of ROS is important for the correct course of various biological processes, for example, the cell cycle, immunological response and the expression of various genes, such as the genes that encode cytokines [33]. The expression of cytokine genes is initiated by transcription factors; NF-κB and AP-1 are regulated by reactive forms of oxygen [10,73]. It is believed that the activation of NF-κB during active muscle work is the basis of the organism’s ability to adapt to exercise [7]. The presence of ROS in the location of the myocytes damage is partially responsible for the reconstruction of the muscle fibers [37].

The ROS, which are generated by phagocytes in muscles fibers during the local inflammatory reaction, are not the only factors that induce the expression of the Il-6 gene. The secretion of myokines in the skeletal muscles is also probably linked with the changes in glycogen and the concentration of calcium ions during exercise [37]. The decrease in the amount of glycogen in skeletal muscles induces the generation of Il-6, simultaneously stimulating the process of glycogenolysis in hepatocytes and lipolysis in adipocytes [6,74]. It has been proven that Il-6 activates membrane glucose transporters [6] and strengthens the process of β–oxidation of fatty acids [74,75]. At the same time, the expression of the Il-6 gene is clearly relative to the concentration of the hydrogen peroxide produced during aerobic metabolism [71]. This fact may explain the tendency for the Il-6 concentration to rise in blood after exercise, which has been observed in our own studies. 

Intensive exercise leads to muscle fiber damage and a local inflammatory reaction. However, regular, properly conducted training generates an anti-inflammatory mechanism; thus, it influences the growth of the organism’s adaptation to larger exercise loads. A disruption of the oxidant–antioxidant homeostasis of the organism during physical effort may strengthen the inflammatory process and the damage of tissues by ROS. A significant increase in the generation of free radicals blocks the proliferation and differentiation of satellite cells in the location of the micro-damage, which as a result disrupts the process of muscle fiber regeneration [5]. 

The mechanisms responsible for the generation of cytokines under physical stress have not been thoroughly analyzed. Some authors prove that oxidative stress is the main factor stimulating their synthesis [37]. The research on stress caused by extremely low temperatures shows that WBC may stimulate the immunological response of the organism [2,24,76].

Exposing the organism to low temperatures seems to act as a considerable boost to leucocyte mobilization. Experimental studies have shown significant changes in leucocyte fractions in healthy men exposed to the temperature of −130 °C, as a WBC, twice, before and after the winter-swimming season [23]. The authors have shown that exposition to cryogenic temperatures causes changes in the percentage of monocytes and granulocytes, depending on the exposure time. An increase in the percentage of granulocytes and decrease in the percentage of monocytes has been reported that did not affect the total number of white blood cells, remaining at a level comparable to the rest period (before the experiment) [23]. The influence of long-term WBC (temperature: −140 °C ± −10 °C) linked with kinesiotherapy on specific lymphocyte subpopulations in peripheral blood has been proven in patients with rheumatoid arthritis (RA) and osteoarthritis (OA) [41]. In the case of patients with RA, on the 7th day of the therapy, an upsurge in the fraction of helper, cytotoxic and suppressor lymphocytes T and lymphocytes B has been shown with statistical importance. A statistically significant increase in natural killer (NK) cells has been observed both in patients with RA as well as with OA. The authors suggest that the immunomodulatory influence of WBC preceding movement therapy is probably related to the limitation of the inhibitory effect of cytokines and prostaglandins on NK lymphocytes, which is observed in the course of chronic inflammatory diseases [41]. 

The change in the inflammatory response of the organism to exercise observed in this study as an effect of cryogenic temperatures may be linked with the activation of the sympathetic nervous system and a secondary increase in the concentration of cortisol and catecholamines in the blood. The expression of α- and β-adrenergic receptors and their bonding with catecholamines may induce or inhibit different pathways of signal transduction. It has been shown that the stimulation of β2-adrenoreceptors manifests itself in the decrease in pro-inflammatory cytokine synthesis and the concurrent stimulation of anti-inflammatory cytokines synthesis [77]. The lower concentrations of IL-1β observed in our own studies in the blood of well-trained athletes after the exercise preceded by WBC (when compared to the exercise without cryostimulation), as well as the tendency for the TNF-α concentration to drop in the blood of sportsmen after the exercise with WBC (when compared to the concentration of the interleukin measured during rest), are probably linked with the generation of catecholamines and the activation of β2-adrenergic receptors induced by cryostimulation [78]. A confirmation of the dampening of the micro-damage-induced inflammatory reaction caused by the exposition to extremely low temperatures is possibly associated with the tendency for the CK activity to drop, which can be observed directly after the procedure, in addition to the slightly lower activity of this enzyme in the blood and the lower concentration of TGF-β1 after exercise preceded by WBC when compared to the control exercise [20,24].

Rhind et al. [79] obtained similar results to those illustrated in this study. The authors noticed an intensification of the expression of the genes that code Il-1β, Il-6 and TNF-α in the case of young volunteers in good physical shape directly after the sports training, while on the other hand, when the training was preceded by the exposure of the organism to low temperatures, a drop in Il-1β and TNF-α expression was observed. Some findings confirm that low temperatures initiate changes in the expression of cytokines linked with non-specific acute phase reactions [3]. It is thought that the reactions between hormones and cytokines that happen during the exposition to cryogenic temperatures may regulate the immunological homeostasis of the organism. Cytokines act as a mediator in the relay of signals between the neuroendocrine and immunological system [77]. Anti-inflammatory activity of long-term WBC has been observed in rugby players [2]. In the blood of these sportsmen, an increase in anti-inflammatory Il-10 has been observed, along with a concurrent drop in the level of pro-inflammatory interleukin 2 and interleukin 8, which shows chemotactic activity towards phagocytes. A reduction in the number of intercellular adhesion molecule-1 (ICAM-1) taking part in the inflammatory response has also been observed [2]. On the other hand, Krueger et al. [80] did not show differences in the IL-6 and IL-10 concentration when compared to high-intensity running followed by 3 min of WBC with a control condition. It is noteworthy, however, that in the quoted article, there was a small number of study participants and there were some methodological differences (in the research experiment), which may have resulted in a difference in the concentration of cytokines (in relation to our own research).

The change in the cytokine profile that has been observed in well-trained athletes after intensive exercise preceded by a single WBC session suggests that even a single exposure to very low temperatures may normalize the body’s inflammatory response to exercise. Statistically significant correlations between the level of cytokines and oxidative stress markers in the blood of the male players of sports clubs, both after leaving the cryochamber and after the exercise preceded by WBC, offer some evidence that reactive forms of oxygen participate in the secretion of cytokines into the blood. Moreover, these findings suggest that preceding exercises with at least a single session of cryostimulation probably prevent muscle inflammation’s negative consequences after intense exercise in well-trained athletes.

### Limitations

This research experiment has potential limitations. The studies were conducted on a specific population of well-trained athletes. Moreover, only male athletes practicing various sports disciplines participated in the study. Therefore, the obtained results cannot be generalized to the whole population, even considering other populations of professional athletes. The lack of variation in the results after exercise with and without WBC is perhaps due to the particular category of subjects used. Furthermore, the parameters of oxidative damage/oxidative stress determined in our own research are limited. Thus, for a better understanding and the possibility of explaining this phenomenon, also in response to the variability in the antioxidant status, it seems necessary to conduct other, more in-depth studies.

## 4. Materials and Methods

### 4.1. Participants 

A number of 32 healthy, professional, male athletes that were players of sports clubs (volleyball, basketball and football) participated in the study during their rest time (regeneration period) following a sports competition (Table 1). The athletes qualified for the study by a physician (specialist in orthopedics and traumatology) and a physiotherapist (specialist in physiotherapy in orthopedics and sports medicine). The eligibility criteria are defined in the table below (Table 4).

Out of the 37 volunteering athletes who offered to participate in the experiment, only 32 passed the qualification process for the study. The reasons for excluding 5 volunteers from the study were most often the following: the presence of musculoskeletal injuries (*n* = 2), consumption of dietary supplements containing antioxidants (*n* = 1), and undergoing physical and wellness treatments (*n* = 2).

### 4.2. Study Design

The study was performed in the “Sports and Recreation Center—Sport Factory” (Bydgoszcz, Poland). The participants were assigned to either submaximal exercise preceded by a WBC procedure or to control exercise (without cryostimulation) in a crossover order. During the first exercise (session 1), the athletes underwent a single WBC procedure, and next (7 ± 2 min after leaving the cryochamber), a 40 min submaximal physical effort with the use of the cycle ergometer Monark 828 E (Monark Exercise AB, Vansbro, Sweden). During the second part of the experiment (session 2), the subjects were exposed to the same exercise (40 min submaximal), except for cryostimulation (control exercise). The interval between sessions was 14 days. Each procedure (the first and the second session) took place at the same time of the day (morning hours). A 5 min warm-up preceded each exercise (during session 1 and session 2) at an intensity of 1.5 W per kilogram of body weight. Immediately after the warm-up, the study participants began submaximal exercise. The burden of the organisms was indicated by the heart contraction incidence, which constituted the physiological criterion of body fatigue. Exercises (throughout both stages of the study) included 40 min of cycling at an intensity corresponding to approximately 85% of their maximum heart rate (HR_max_). HR values were monitored continuously, and the workload (wattage) was adjusted every 5 min as necessary to maintain 80–85% of HR_max_. The Polar H7 Heart Rate Sensor (Polar Electro Oy, Helsinki, Finland) equipped with a PC was used to supervise the HR. The mean power of the first exercise was about 180 W with a pedaling cadence of about 80 rpm, while the second exercise (control) was about 175 W with the same pedal frequency. The safety and course of the study (during session 1 and session 2) were supervised by a physician experienced in performing this type of research experiment.

The studies were carried out in accordance with the Declaration of Helsinki. Written consent was obtained from each subject, and the Bioethical Committee approved the study at the Collegium Medicum of Nicolaus Copernicus University, Poland. All participants were informed about the purpose and the course of the research, as well as the option of resignation from participation in the study at any stage. The health and safety of the athletes during each phase of the study were supervised by a physician researcher, whose qualifications were verified before granting permission to conduct the study by the Bioethics Committee. This study was registered in the Australian New Zealand Clinical Trials Registry (ANZCTR) under the registration number ACTRN12618001711202.

#### The WBC Procedure

The cryogenic chamber (Arctica, Metrum CryoFlex, Blizne Łaszczyńskiego, Poland) was cooled with the use of synthetic vapors and liquid air with 22% ± 2% of oxygen, which resulted in improved safety. The cryogenic chamber was located approximately 2.5 m below the floor surface, allowing the cold retention phenomenon to be applied. The stairs used by every participant when entering the study room were used as a mild adaptation zone. At the bottom of the stairs, there was a hallway where the temperature was kept at –60 °C. Double swing doors separated the hallway and the main chamber. Throughout the process, the temperature remained at −130 °C. In order to protect the body against frostbite, the athletes were required to wear safety suits before entering the cryochamber. Dust masks were used to protect the airways. All participants received information regarding proper behavior during the process, i.e., taking slow and shallow breaths (short nasal inhalation and longer oral exhalation) and slow circular walks. The duration in the cryochamber did not exceed 2 min. Before entering the main chamber, every participant was subjected to a 10 or 20 s adaptation procedure in the hallway at the temperature of −60 °C. It was only after the positive result of the medical examination that the athletes were accepted for the WBC.

### 4.3. Baseline Examination

The anthropometric evaluation of the participants included the following measurements: body mass (BM, kg), body height (BH, cm) and body mass index (BMI, kg/m^2^) with the use of medical scales with a telescopic measuring device (HM 201M with BMI function, CHARDER MS 4971, JAWAG, Bilcza, Poland). BM measurements were performed twice prior to each experimental session (before session 1 and before session 2) and the BH measurements were taken only once, before the start of the study (prior to session 1).

Aerobic capacity was assessed indirectly based on the PWC170 test results [81]. The primary purpose of this test was to determine the index of total work (an estimated power of effort) performed at the heart rate (HR) of 170 beats per minute (bpm) using extrapolation, and to indirectly estimate VO_2_ max. The subjects refrained from any physical training or activity at least one day prior to the test. Before the test, body mass was measured. The primary purpose of this test was to determine the index of total work (an estimated power of effort) performed at the heart rate (HR) of 170 beats per minute (bpm) using extrapolation, and to indirectly estimate VO_2_ max. The PWC170 test was performed using a cycloergometer (Monark Ergomedic 828 E, Vansbro, Sweden). After a 5 min warm-up, the players performed 2 standard submaximal 5 min cycloergometer tests, where the load in the second test was increased so that the HR of 170 bpm was not exceeded. The intensity of these tests was adjusted for individual players so that their HR during the physiological steady state ranged between 120 and 150 bpm. Their heart rate was determined using the Polar H10 heart rate monitor (Polar Electro Oy, Helsinki, Finland). The mean value of the HR was registered at the last minutes of each 5 min stage [81]. The test result was calculated using the following formula: PWC170 = P1 + (P2 − P1)/(170 − HR1) (HR2 − HR1), where P1 represents the power of the first exercise test, P2 is the power of the second exercise test, HR1 is the HR during the first exercise test and HR2 is the HR during the second exercise test. The PWC170 index was calculated in Watts and Watts/kg and then VO_2_ max in L/min and in mL/kg/min, using Karpman’s formula, which is as follows [82]: VO_2_ max = (10.2 × PWC170) + 1240, where 10.2 and 1240 are dimensionless constants. The PWC170 test was performed twice, before each experimental session (before session 1 and before session 2.).

The Borg Category Ratio-10 (CR10) scale was used to identify the subjective assessment of the intensity of exercise, where 0 means “no exertion at all” and 10 means the “maximal” level of exertion [83]. Each study participant defined their perceived exertion (RPE) immediately after the exercise preceded by WBC and after the control exercise (without WBC).

The International Physical Activity Questionnaire—Short Form (IPAQ—SF) was used to characterize the level of physical activity undertaken by the athletes in the period preceding the start of the study. IPAQ is formulated in MET-min/week. One MET equals 3.5 mL O_2_/min/kg and represents the baseline oxygen consumption [84]. The athletes answered the questions included in the questionnaire immediately after the end of the sports season and on the first day of the experiment (the 15th day of rest following sports competitions), before the start of the study.

### 4.4. Blood Sampling

Blood samples were collected by a physician (with appropriate qualifications and experience) and then transported in a travel refrigerator (at 4 °C ± 1 °C) by another scientist (biochemist, medical analyst) to the Biochemistry and Immunology Laboratory in the Department of Medical Biology and Biochemistry, Ludwik Rydygier Collegium Medicum in Bydgoszcz, Nicolaus Copernicus University, Torun, Poland.

During the session 1, blood samples were taken the following three times: first, before entering the cryochamber, then, no sooner than at the beginning of the exercise (3 ± 2 min after leaving the cryochamber), and finally, after submaximal exercise using the cycloergometer. Blood samples were taken once during session 1 (control phase), i.e., after the control exercise, 2 weeks following the cryochamber stimulation. We established the investigated parameters using the blood samples obtained from antecubital veins. Blood from the vein was placed into the following two test tubes: the first one contained an anticoagulant, dipotassium edentate (K_2_EDTA, ethylenediaminetetraacetic acid dipotassium salt), taken for a whole blood test, and the second one was dry, to obtain blood serum.

### 4.5. Biochemical Analysis

The activity of SOD, GPx, CAT and CK was examined in the erythrocytes. The concentration of TBARS was marked in plasma and erythrocytes, while CD and vitamin A and E levels were measured in the plasma. Moreover, the concentration of IL-1β, IL-6, TNF-α, TGF-β1 and LOOH was determined in blood serum. The chemical factors used for testing (CD and TBARS level, vitamin A and E concentration, as well as SOD, CAT and GPx activity) were obtained from Sigma-Aldrich Corporation LLC. (Saint Louis, MO, USA) and Avantor Performance Materials Poland S.A. (Gliwice, Poland). The measurements of sample absorbance were taken with a CARY 1E UV–Vis spectrophotometer supported by Cary WinUV software (version number: 3.00(182), Varian, Palo Alto, CA, USA).

#### 4.5.1. Determination of the TBARS Concentration

The concentration of TBARS was assayed using a manual procedure both in the erythrocytes and in plasma [85]. The method involves the formation of a complex between the products of lipid peroxidation and thiobarbituric acid (TBA) (at the temp. of 100 °C). TBA was used for testing the lipid peroxidation products. The most essential product of lipid peroxidation that reacts with thiobarbituric acid is malondialdehyde (MDA). The TBARS level in plasma was shown in nmol of MDA/mL. The erythrocytes levels were determined in nmol of MDA/gHb. We added 0.5 mL of hemolysate or 0.5 mL of plasma to 4.5 mL of the reaction mixture (0.375% of TBA and 15% of trichloroacetic acid (TCA) in 0.25 M hydrogen chloride (HCl). The solution of 0.01% 3.5-dibutyl-4-hydroxy toluene (BHT) (inhibitor of the lipid peroxidation process) was added to avoid forming lipid peroxidation products. To optimize the reaction conditions, a water bath at 100 °C was used to incubate the mixtures for 20 min. Afterward, they were cooled and centrifuged at 2000× *g* at 4 °C for 15 min. The supernatant extinction was measured at the wavelength of 532 nm, with respect to the reaction mixture incubated in the same conditions [85]. The intra-assay and the inter-assay coefficient of variations as % CV were 6.5–10.2% and 8.6–11.9%, respectively.

#### 4.5.2. Determination of CD Level

The CD were formed during the lipid peroxidation due to the rearrangement of double bonds after the detachment of hydrogen atoms from the remaining polyunsaturated fatty acids. During this reaction, the characteristic absorption peak at the wavelength of 233 nm was observed. To determine the CD level, 0.5 mL of chloroform was mixed with 0.5 mL of the plasma and centrifuged; this was followed by placing 0.1 mL of the solution into a clean test tube. Then, the samples were vaporized in a nitrogen atmosphere and dissolved in cyclohexane. The absorbance readings were taken at the wavelength of 233 nm [86]. The CD level was presented in the absorbency units per ml of plasma (Abs./mL). The sensitivity of this assay was up to a few nanomoles (2–3 nmoles). The assays had a within-run CV range from 5.7 to 7.3% and a between-run CV from 4.6 to 8.3%.

#### 4.5.3. Determination of SOD, GPx and CAT Activity

SOD activity was evaluated in erythrocytes hemolysates with the method based on the enzyme slowing down the reaction of the auto-oxidation of adrenalin to adrenochrome in an alkaline medium [87]. The unit of SOD activity is the quantity of the enzyme that slows down the reaction by 50% at a maximum increase in the absorption of 0.025 U/min on a rectilinear section of adrenochrome formation. GPx activity was determined in erythrocytes by identifying the differences in absorption that resulted from the change in the reduced form of NADPH into an oxidized form [88]. NADPH acted as a coenzyme in the reaction of the reduction of glutathione disulfide catalyzed by glutathione reductase. The obtained oxidized glutathione was the product of the reaction catalyzed by GPx. Hydrogen peroxide was the substrate. The unit of SOD and GPx activity was U/gHb. The catalyzed activity was determined by using the Beers and Sizer method [89], which is based on measuring the absorbance decrease in hydrogen peroxide decomposed by CAT, measured at a wavelength of 240 nm. The unit of CAT activity was IU/g Hb. The manual procedure performed the determination of antioxidant enzymes (SOD, GPx and CAT) activity. The reagents were provided by Sigma (Sigma-Aldrich Co. LLC, Saint Louis, MO, USA) and Avantor Performance Materials Poland S.A. (formerly POCH S.A.) (Gliwice, Poland). The optical density was evaluated using the “Cary 100” spectrophotometer (Varian, Palo Alto, CA, USA).

#### 4.5.4. Determination of A and E Vitamin Concentration and CK Activity

The concentration of vitamins A and E was marked using the high-performance liquid chromatography (HPLC) setup. To obtain the relevant data, a 20 µL solution of the internal model (retinol acetate for vitamin A; α-tocopherol acetate for vitamin E) was added to 200 µL of blood plasma. Next, 800 µL of acetonitrile was added and shaken to enable protein denaturation. The obtained suspension was subjected to whirling; later, the supernatant was filtered in the SPE system (Captiva 2 µL). About 4 mL of hexane was added into the test tubes for extraction. After this stage, the samples were shaken, whirled and frozen for approximately 45 min at a temperature of −80 °C. Next, the hexane fraction containing vitamin A and vitamin E was decanted into test tubes and vaporized until dry in the nitrogen atmosphere at 40 °C. The obtained sample was dissolved by adding a 100 µL phase, then mixed with the use of ultrasounds and 10 μL of the sample was given to the sample loop of the analyzer using a Kinetex C18 column (2.6 μm). HPLC separation was carried out using a mobile phase of 95% *v*/*v* acetonitrile and 5% *v*/*v* methanol and a flow rate of 2 mL/min. The vitamins were detected using the UV–Vis detector at the wavelength of λ = 292 nm. Star Chromatography Workstation software (version number: 6.41, Varian, Palo Alto, CA, USA) was used to mark the concentration of the studied vitamins, and the results were given in mg/L.

To evaluate total CK activity, ready-made reagent kits (Alpha Diagnostics, Warsaw, Poland) were used. The assay was based on the Rosalki [90] and Szasz et al. [91] procedure. The activity of CK was given in IU/l.

#### 4.5.5. Determination of the LOOH Concentration

The total concentration of lipid peroxides (LOOH) in blood serum was determined using the enzyme-linked immunosorbent assay (ELISA) by the instructions provided by the producer. The commercial kits for this test were produced by Biomedica Poland Sp. z o. o. (Piaseczno, Poland). The total concentration of lipid peroxides in blood serum was given in mol/L. The limit of detection for the LOOH test was 7 mol/L.

#### 4.5.6. Determination of the Cytokine Concentrations

The concentration of the cytokines Il-1β, Il-6, TNF-α and TGF-β1 was determined in blood serum using four different solid phase ELISA kits. The measurements were taken in accordance with the instructions provided by the manufacturer (Biomedica Company, Piaseczno, Poland). The method was based on the binding of cytokines by monoclonal antibodies for a given cytokine. The antibodies were absorbed into microwells. During the first incubation, a cytokine bound to the antibodies present in the sample was absorbed into the microwells. After washing, a biotin-conjugated monoclonal antibody, specific for the cytokine, was added. During the second incubation, this antibody was bound to the immobilized cytokine captured during the first incubation (by the first antibody). Following the incubation, during the wash stage, the unbound biotin-conjugated antibody was removed with a microplate washer (Thermo Fisher Scientific Oy, Vantaa, Finland). Once the second antibody was removed, streptavidin protein conjugated to horseradish peroxidase (Streptavidin-HRP) was added and connected to the biotin-conjugated antibody to complete the four-layer sandwich. After the third incubation and washing, the purpose of which was to remove all the unbound enzymes, a substrate solution reactive to HRP, which was activated by the bound enzyme to produce color, was transferred to the wells. A colored complex was created in proportion to the amount of cytokine present in the sample or standard. The reaction ended with adding acid and measuring the absorbance at λ = 450 nm. The concentration of the tested antigen was determined based on a model curve. The intensity of the colored product was proportional to the cytokine concentration found in the original specimen. The amounts of the marked cytokines were expressed in pg/mL (level of: Il-1β, Il-6, TNF-α) and in ng/mL (level of TGF-β1). The immunoenzymatic tests and the calibrations were performed in accordance with the study protocols included with the test kit. The sensitivity of a given method depended on the applied calibrators. The detection limits for the cytokines were as follows: IL-1β—0.32 pg/mL, IL-6—0.92 pg/mL, TNF-α—4 pg/mL and TGF-β1—0.02 ng/mL.

### 4.6. Statistical Analysis

The outcome is displayed as ±SD (standard deviations). The statistical analysis was based on repeated measures ANOVA with Tukey’s HSD test. Before running ANOVA with repeated measurements, tests were run on model assumptions, and the Kolmogrov–Smirnov test’s results met the normal distribution requirements. Moreover, Levene’s test confirmed the homogeneity of covariance. The ANOVA effect size in the sample was determined by eta-square values (η^2^), where 0.01 indicates a small effect, 0.06 is an average effect, and 0.14 is a large effect. Correlation matrices were used for estimating the reliance on the evaluated data. Spearman’s test was used for estimating a statistical hypothesis of the importance of the correlation coefficient (r). The differences with a significance level < 0.05 were significant from a statistical point of view. The statistical results were prepared using the STATISTICA 12.0 (StatSoft, Krakow, Poland) statistical software package.

## 5. Conclusions

The changes in the cytokine concentration in well-trained male athletes confirm that body exposure to extremely low temperatures before exercise regulates the inflammatory reaction course and secretion of cytokines during exercise. The influence of WBC on the regulatory mechanisms of cytokine secretion during excessive exercise is does not directly depend upon the oxidative–antioxidative reaction course. A single session of WBC in the case of professional, male athletes does not significantly affect the level of oxidative stress indicators. However, the correlations shown between the marked parameters and lower CAT activity in the athletes’ blood after the exercise preceded by WBC indicate a beneficial effect of cryogenic temperatures on the oxidant–antioxidant balance.

## Figures and Tables

**Table 1:** Selected physical characteristics of the subjects (*n* = 32).

Parameter	Mean ± SD
Age (year)	25.2 ± 2.37
Height (cm)	186.0 ± 5.02
BM ^1^ (kg)	82.56 ± 14.9
BM ^2^ (kg)	80.8 ± 12.3
BMI ^1^ (kg/m^2^)	23.1 ± 0.18
PWC170 ^1^ (W)	320.3 ± 70.9
PWC170 ^2^ (W)	321.9 ± 70.8
PWC170 ^1^ (W/kg)	3.98 ± 1.17
PWC170 ^2^ (W/kg)	4.05 ± 1.08
VO_2_ max ^1^ (mL/kg/min)	57.05 ± 2.9
VO_2_ max ^2^ (mL/kg/min)	56.09 ± 3.05 *
Borg CR10 ^I^	6.12 ± 0.93
Borg CR10 ^II^	6.06 ± 0.788 ^#^
Training experience (year)	13.4 ± 1.38

BM ^1^—body mass measured prior to session 1, BM ^2^—body mass measured prior to session 2, BMI ^1^—body mass index calculated from BM ^1^, PWC170 ^1^—the result of the PWC170 test performed prior to session 1, PWC170 ^2^—the result of the PWC170 test performed prior to session 2; VO_2_ max ^1^—maximum oxygen consumption calculated from the PWC170 test result performed before session 1, VO_2_ max ^2^—maximum oxygen consumption estimated from the PWC170 test result performed before session 2; Borg CR10 ^I^—Borg Rating of Perceived Exertion Scale after session 1, Borg CR10 ^II^—Borg Rating of Perceived Exertion Scale after session 2; * the difference in comparison with the value before session 1 (*p* > 0.05); ^#^ the difference in comparison with the value after session 1 (*p* > 0.05).

**Table 2:** Concentration of conjugated dienes (CD), lipid peroxides (LOOH), thiobarbituric acid reactive substances (TBARS), vitamins (vit.) A and E, as well as superoxide dismutase (SOD), catalase (CAT) and glutathione peroxidase (GPx) activity in sportsmen’s blood after whole-body cryostimulation (WBC) and/or submaximal exercise.

Parameter	Research Date			
	Before the Start of the Study (before WBC)	Following WBC	Following Exercise Preceded by WBC (WBC Exercise)	Following Exercise without WBC (Control Exercise)
	Mean ± SD			
CD in plasma (10^−2^ Abs./mL)	8.14 ± 3.25	6.6 ± 3.20	6.83 ± 3.27	7.77 ± 3.13
TBARS in erythrocytes (nmol MDA/g Hb)	42.87 ± 15.69	38.7 ± 13.01	37.78 ± 12.78	40.14 ± 11.15
TBARS in plasma (10^−2^ nmol MDA/mL)	39.97 ± 15.07	38.34 ± 15.04	34.37 ± 8.7	40.06 ± 8.21
LOOH in serum (mol/L)	188.0 ± 78.89	183.0 ± 80.11	209.0 ± 81.88	227.1 ± 74.54
vit. A in plasma (mg/L)	234.9 ± 83.96	224.2 ± 89.09	227.5 ± 87.14	219.2 ± 83.22
vit. E in plasma (mg/L)	701.5 ± 236.6	684.3 ± 263.8	678.6 ± 239.6	585.6 ± 253.7
SOD (U/g Hb)	1263.0 ± 250.6	1315.7 ± 288.8	1366.5 ± 273.5	1407.0 ± 305.5
CAT (10^4^ IU/g Hb)	52.25 ± 19.53	51.87 ± 19.43	51.09 ± 20.25	67.14 ± 11.6 ^aa bb ccc^
GPx (U/g Hb)	3.87 ± 1.62	3.81 ± 1.67	3.88 ± 1.68	4.88 ± 1.58

^a^ Statistically significant difference in comparison with the activity measured before WBC (^aa^ *p* < 0.01); ^b^ statistically significant difference in comparison with the activity measured after WBC (^bb^ *p* < 0.01); ^c^ statistically significant difference in comparison with the activity measured after the exercise associated with WBC (^ccc^ *p* < 0.001).

**Table 3:** The creatine kinase (CK) activity, interleukin-1β (IL-1β), interleukin-6 (IL-6), tumor necrosis factor alpha (TNF-α) and transforming growth factor beta1 (TGF-β1) concentration in sportsmen’s blood after single whole-body cryostimulation (WBC) and/or submaximal exercise.

Parameter	Research Date			
	Before the Start of the Study (before WBC)	After WBC	After Exercise Preceded by WBC (WBC Exercise)	After Exercise without WBC (Control Exercise)
	Mean ± SD			
CK (IU/L)	204.9 ± 96.74	181.4 ± 89.37	203.4 ± 82.17	233.8 ± 79.96
IL-1β (pg/mL)	6.72 ± 3.26	6.87 ± 2.65	4.83 ± 1.91	9.93 ± 3.73 ^aa bb ccc^
TNF-α (pg/mL)	10.93 ± 5.29	10.08 ± 5.24	7.78 ± 2.47	10.63 ± 4.58
IL-6 (pg/mL)	4.75 ± 2.36	2.27 ± 1.05 ^aa^	4.42 ± 1.01 ^b^	5.95 ± 2.45 ^bbb^
TGF-β1 (ng/mL)	31.23 ± 15.61	34.37 ± 14.65	33.59 ± 16.05	37.39 ± 13.39

^a^ Statistically significant difference compared with the concentration measured before WBC (^aa^ *p* < 0.01); ^b^ statistically significant difference compared with the concentration measured after WBC (^bbb^ *p* < 0.001; ^bb^ *p* < 0.01; ^b^ *p* < 0.05); ^c^ statistically significant difference compared with the concentration measured after the exercise associated with WBC (^ccc^ *p* < 0.001).

**Table 4:** Eligibility criteria.

Inclusion Criteria
No injury (musculoskeletal and/or other systems) or illness that could affect the course or outcome of the study; No medication (one month before beginning the study as well as during the experiment); No dietary supplements or herbal preparations containing antioxidants or anti-inflammatories (one month prior to beginning the study as well as during the experiment); No physical or wellness treatments (one month before beginning the study as well as during the experiment).
Exclusion Criteria
Presence of injuries (musculoskeletal and/or other systems) or conditions that could affect the course or outcome of the study; Taking any medications (one month before beginning the study as well as during the experiment); Consumption of dietary supplements and herbal preparations containing antioxidants or anti-inflammatories (one month before beginning the study as well as during the experiment); Receiving physical therapy and wellness treatments (one month before beginning the study as well as during the experiment); Use of stimulants (e.g., smoking).

## Data Availability

The data presented in this study are available upon request from the corresponding author. The data are not publicly available due to privacy or ethical restrictions.
